# Effect of Frailty on Functional Gain, Resource Utilisation, and Discharge Destination: An Observational Prospective Study in a GEM Ward

**DOI:** 10.1155/2014/357857

**Published:** 2014-02-17

**Authors:** Sujatha Kawryshanker, Warren Raymond, Katharine Ingram, Charles A. Inderjeeth

**Affiliations:** ^1^Sir Charles Gairdner Hospital, Hospital Avenue, Nedlands, WA 6009, Australia; ^2^Osborne Park Hospital, Osborne Place, Stirling, WA 6021, Australia; ^3^The University of Western Australia, 35 Stirling Highway, Crawley, WA 6009, Australia

## Abstract

*Background*. A geriatric evaluation and management unit (GEM) manages elderly inpatients with functional impairments. There is a paucity of literature on frailty and whether this impacts on rehabilitation outcomes. *Objectives*. To examine frailty score (FS) as a predictor of functional gain, resource utilisation, and destinations for GEM patients. *Methods*. A single centre prospective case study design. Participants (*n* = 136) were ≥65 years old and admitted to a tertiary hospital GEM. Five patients were excluded by the preset exclusion criteria, that is, medically unstable, severe dementia or communication difficulties after stroke. Core data included demographics, frailty score (FS), and functional independence. *Results*. The mean functional improvement (FIM) from admission to discharge was 11.26 (95% CI 8.87, 13.66; *P* < 0.001). Discharge FIM was positively correlated with admission FIM (*β* = 0.748; *P* < 0.001) and negatively correlated with frailty score (*β* = −1.151; *P* = 0.014). The majority of the patients were in the “frail” group. “Frail” and “severely frail” subgroups improved more on mean FIM scores at discharge, relative to that experienced by the “pre-frail” group. *Conclusion*. All patients experienced functional improvement. Frailer patients improved more on their FIM and improved relatively more than their prefrail counterparts. Higher frailty correlated with reduced independence and greater resource utilisation. This study demonstrates that FS could be a prognostic indicator of physical independence and resource utilisation.

## 1. Background

Hospitalisation can result in a decline in patients' functional status, mental status, continence, and ability to accomplish activities of daily living [1]. Australia's population is ageing as a result of sustained low fertility and increasing life expectancy [2]. As healthcare costs are high, the need to decrease negative outcomes in personal health is an important social issue. The Australian Bureau of Statistics predicts that the proportion of Australians over 65 years could increase from 13% to 25% between 2007 and 2056. Therefore, it is of paramount importance to healthcare providers to identify, screen, and stringently manage older persons at the risk of physical deterioration during hospitalisation [3].

There is now increasing evidence that frailty is a geriatric syndrome, indicative of progressive decline, increased vulnerability to stressors, and increased risk of adverse health outcomes [4]. Frailty, once termed “failure to thrive” in older adults, is also a condition that is predictive of injurious falls, impaired mobility, worsening disability, hospitalisation, institutionalisation, and death [5]. Frailty, a multidimensional geriatric syndrome, may be measurable using appropriate scoring instruments [6]. However, the reliability of the scoring instruments has not yet been proven to be effective in clinical practice. Currently there is limited validation of the tools which assess frailty.

Interventions that can improve outcomes of acute hospitalisations in older persons include the multidisciplinary care model, elder-friendly environment, and facilitated exercise programs [7, 8]. Geriatric evaluation and medical management (GEM) is a model which provides rehabilitation and care of older persons. GEM comprises an interdisciplinary team trained to manage the medical, psychological, and functional issues in older persons [9]. GEM is a ward model of comprehensive geriatric assessment (CGA), combined with rehabilitation, development of care plans, and comprehensive discharge planning [10]. Available research evidence strongly supports the benefits of this model in the acute sector as a way to reduce hospital costs through reduced length of stay and readmissions. The CGA model aims to deliver improvement in the patients' quality of life, through promoting independence and reducing nursing home admission [10–12]. Meta-analysis of randomised control trials [13] concluded that patients were also less likely to die or experience deterioration in the CGA group. Rehabilitation in GEM is usually short term. Therefore, early identification of appropriate inpatients for GEM is essential to maximise effective and efficient use of the unit. Our study aimed to demonstrate the efficacy of a GEM rehabilitation intervention for patients' who experience an increased state of frailty related to a medical or surgical admission. Our study measured changes in the functional improvement measurement (FIM) scores at admission and discharge against frailty status of patients at discharge (based on the Edmonton Frailty Scale (EFS)). It is anticipated that our research findings may provide a guide for the selection of patients deemed suitable for multidisciplinary GEM intervention, to reduce LOS and healthcare costs by applying a short term, multidisciplinary intervention to patients who are expected to return to independent living after rehabilitation.

## 2. Methods

### 2.1. Setting and Sample

This was a prospective observational case study of patients admitted to a geriatric evaluation medical unit (GEM) at a tertiary teaching hospital in Western Australia between 15th of February and 15th of May 2012.

Participants were older persons (men and women aged 65 years or more) admitted to the unit. GEM unit, a short stay in patient rehabilitation, incorporates a geriatrician, advanced trainee (medical registrar), nursing staff, and multidisciplinary allied health professionals. Eligible participants were inpatient referrals from medical and surgical specialties. These referrals were assessed by a single geriatric advanced trainee (the researcher) for suitability of admission to GEM and then enrolled into the study. Verbal informed consent was obtained from each participant.

The inclusion criteria were adult persons aged 65 years or older, predicted discharge to usual place of residence within 14 days, and the need for multidisciplinary rehabilitation. The exclusion criteria included patients not suitable for GEM including a predicted LOS greater than 14 days, unable to participate in effective rehabilitation due to severe dementia or unstable medical conditions. Post-stroke and readmitted patients, who can confound the findings through double counting, were excluded.

### 2.2. Demographics

All demographic details including name, residence, and age were obtained by a single researcher to ensure consistency.

### 2.3. Geriatric Syndrome (GS)

The presence of GS was based on participant or carer report and clinical diagnosis. These include cognitive impairment, falls in past 6 months, delirium, urinary and faecal incontinence, and history of depression.

### 2.4. Measurements and Procedures

The Western Australian Department of Health has paid for the right to use the FIM tool in clinical settings. Functional independence measure (FIM) [14] was measured by a single senior occupational therapist (OT) at admission as a baseline and within 48 hours of discharge as an outcome measure. The FIM is an 18-item scale rating activities of daily living and independence in the domains of self-care, sphincter control, mobility, locomotion, communication, and social cognition. The total FIM score has a range from 18 (dependent) to 126 (fully independent). FIM is probably the most widely used generic disability measure [15]. It was developed between 1984 and 1987 and it contains more items than the Barthel Index (BI) [15], such as cognitive items, and has more response categories. It is composed of 13 motor tasks and 5 cognitive tasks (considered basic activities of daily living). Tasks are rated on a 7-point ordinal scale that ranges from total assistance (or complete dependence) = 1 to complete independence = 7. Participants receive multidisciplinary intervention as the usual care of GEM during the stay. Functional outcome assessed by FIM change is defined as the difference between a patient's FIM score at admission and discharge.

Frailty score was measured within 48 hours of discharge from the GEM unit to group patients based on their usual frailty severity. Frailty was measured using the definition of the frailty syndrome by Edmonton Frailty Scale (EFS) [16]. It is a validated brief and user-friendly screening scale for frailty in older patients in both inpatient and outpatient settings [17]. The EFS [17] samples 10 domains; the maximum score is 17 and represents the highest level of frailty. Two domains are tested using performance-based items: the clock test for cognitive impairment and the “timed get up and go” for balance and mobility. The other domains are mood, functional independence, medication use, social support, nutrition, health attitudes, continence, burden of medical illness, and quality of life.

### 2.5. Primary Outcome Measures

The functional independence measure (FIM) was used to assess participants' functional improvement following GEM management at the time of discharge.

Frailty score was measured following recovery from acute episode of care at the end of GEM stay.

### 2.6. Secondary Outcome Measures

Discharge destination is the increase in resource utilisation on the health and welfare system based on discharge destination.

The number of specialist referrals made per patient was also collected as a surrogate marker of complexity/expense of care.

### 2.7. Ethics

Ethical approval was obtained from local research ethics committee (QIA no. 3036).

### 2.8. Analytical Approach

The data was entered onto an excel spread sheet during the data collection period. The statistical analysis was performed using SPSS Version 21.1. Descriptive statistics including percentages, mean, and standard deviation were calculated. Data was expressed as mean ± standard deviation. The study population was divided into prefrail (EFS 0–5), frail (6–9), and severely frail (10–17). Important characteristics were compared using the two-tailed Student's *t*-test. Pearson's correlation was used to analyse the associations between parameters including frailty score, functional gain, and discharge domicile. The value *P* ≤ 0.05 was considered statistically significant. Bivariate analyses with patient outcome were assessed by ANOVA for continuous variables and Chi-square for categorical variables.

To maximise the power of the correlations with the frailty score and FIM difference, categorical analyses were performed. Paired sample *t*-test was used to compare admission and discharge FIM as it is the same sample just measured at two different time points.

## 3. Results

The total number of eligible patients was 131. Five patients were excluded due to dementia, unstable medical conditions, and/or severe post-stroke syndrome. Verbal consent was obtained. Interpreters were used for patients with language barrier.

The mean age of the 131 patients was 82.8 (±8.4) years; 61% were females and 63% were aged above 80. Mean frailty score was 7.6 (±2.9). This indicates that the majority of patients were in the frail category at baseline. Frailty score data was analysed in three groups, that is, as pre-frail (0–5), frail (6–9), and severely frail (10–17). With data from 131 patients assessed, approximately 26% (34/131) were severely frail, 46% (61/131) frail, and 24% (32/131) were pre-frail. The characteristics of the patients recruited to the study are shown in Table 1, classified by frailty status.

### 3.1. Geriatric Syndromes

In the study cohort group, 88.5% of the patients were found to have a geriatric syndrome. The highest prevalent syndrome was falls (74%) followed by dementia (61.8%) (Table 1).

### 3.2. Physical Function at Baseline and Discharge

Functional independence measure (FIM) was used to measure improvements in a patient's physical function. In our cohort the mean admission FIM was 83.1 (±20.4) and mean discharge FIM 94.4 (±20.1). A paired *t*-test was used to compare the mean functional gain (FIM) between admission and discharge; the mean gain in FIM was 11.26 (95% CI 8.87, 13.66) and was statistically significant with *P* < 0.001.

**Figure 1:**
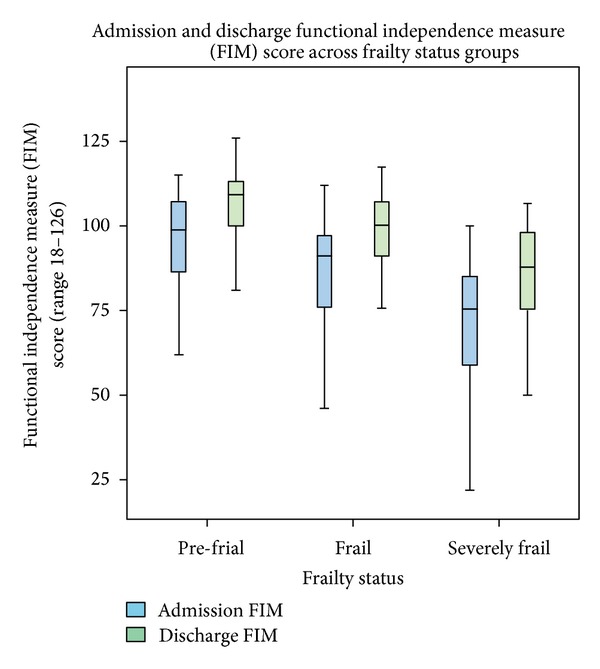
Admission and discharge FIMs against frailty status.

We investigated the changes in FIM at admission and discharge across frailty status groups. All frailty status groups experienced an increase in FIM at discharge. Frail participants had the greatest change in mean FIM, with an admission FIM of 84.3 (±19.3) and a mean discharge FIM of 96.7 (±17.1). Despite a lower mean FIM at discharge than the pre-frail group, the frail group derived a relatively greater functional benefit from the intervention with a mean Difference in FIM (calculated by discharge FIM minus admission FIM) of 12.4 (±11.1) compared with 9.9 (±6.9) for the Pre-Frail group, and 10.5 (±20.9) for the severely frail group (see Table 2 and Figure 1). Pre-frail individuals, that is, those patients with the lowest frailty score, possessed the highest mean admission FIM scores 95.7 (±13.7) and the highest mean discharge FIM of 105.7 (±12.3). The mean FIM gain was the lowest in this cohort. It is likely that this relates to better baseline functional status and thus lower potential for absolute gains. The functional gain was also lower in the severely frail group (lowest mean admission FIM and highest mean frailty score). This may reflect greater physical impairment prior to receiving the intervention with lower reversibility of physical and functional impairment, that is, trough effect.

Frailty score and admission FIM have an inverse relationship (Pearson correlation of −0.456, *P* < 0.001) inferring a moderate negative correlation between frailty score (higher score identifies worsening frailty) and admission FIM (higher score indicates good functional ability). Frailty status was based on cutoffs derived from the frailty score of the patient on discharge. Pre-frail individuals had a mean admission FIM 95.7 (±13.7); frail individuals had a mean admission FIM being 84.26 (±19.3); and severely frail individuals had a mean admission FIM being 70.24 (±20.1). This illustrates the correlation between FIM on admission and frailty status and predicts functional improvement on discharge. This correlation infers the translation of the FIM on admission score as potential categorical tool for clinicians to streamline patients into frailty groups with the objective of assigning specific frailty-level rehabilitation interventions and goals.

A one-way ANOVA was conducted to determine the difference between mean admission FIM across frailty status groups. A between groups *F* value of 16.78 with df = 2 and *P* < 0.001 signifies that there were differences between the mean admission FIM across frailty status groups. Multiple comparisons between frailty status groups for mean admission FIM were assessed with the Bonferroni method. The pre-frail and frail groups had a difference in mean admission FIM of 11.5 (1.8, 21.6), *P* = 0.014. The pre-frail and severely frail groups had a difference in mean admission FIM of 25.5 *P* < 0.001; frail and severely frail groups had a difference in mean admission FIM of 14.02 (4.77, 23.26), *P* = 0.001.

A one-way ANOVA was conducted to determine the difference between mean discharge FIM across frailty status groups. A between groups *F* value of 17.55 with df = 2 and *P* < 0.001 infers that the mean discharge FIM scores are different and with high statistical significance. Multiple comparisons between groups using the Bonferroni method demonstrate that pre-frail and frail groups have a mean difference of 9.03 (−0.45, 18.51), *P* = 0.067, pre-frail and severely frail groups have a mean difference of 24.96 (14.44, 35.47), *P* < 0.001, and the frail and severely frail groups have mean difference in discharge FIM of 15.93 (6.88, 24.98) *P* < 0.001.

Data suggests that the relative improvement was similar in the 3 groups, that is, ±10–12 points. The severely frail group had a mean admission FIM of 70.2 (±20.1) and the lowest mean discharge FIM of 80.7 (±22.8). This implies that although improved, the severely frail patients who entered the inpatient unit with a lower functional status remained functionally impaired at discharge, relative to the pre-frail and frail groups (Figure 1). However, it may be argued that frailer individuals experienced better functional improvement after GEM, but not enough to improve their baseline dependence category, that is, threshold effect and a limited capacity for functional improvement based on a prior physical impairment that was acutely worsened due to admission diagnosis, that is, FIM improvement (limited) but no change in frailty status.

A linear regression was conducted to determine the confounding influence of baseline data on discharge FIM. This investigated the following independent variables including: gender, age, frailty score, admission functional improvement measure (FIM), geriatric syndrome, falls, dementia, other geriatric syndromes, and specialist referrals. The model had an adjusted *R* ^2^ value of 0.606 signifying that the independent variables accounted for 60.6% of the variation in the dependent variable, discharge FIM. The coefficients that exhibited statistical significance included the frailty score at discharge (*P* = 0.045), admission FIM (*P* < 0.001), and specialist referrals (*P* = 0.038). Falls (*P* = 0.059), age (*P* = 0.706), and gender (*P* = 0.401) variables were not statistically significant. In the original regression analysis they were removed in a refined stepwise regression which also included admission FIM, frailty score, and Specialist Referrals. The final stepwise regression model had a robust adjusted *R* ^2^ of 0.607 which infers that the independent variables (admission FIM, frailty score, and specialist referrals) in the regression model account for 60.7% of the variance in discharge FIM. The admission FIM coefficient was statistically significant and had a *β* = 0.748 effect on discharge FIM (*P* < 0.001); frailty score coefficient was statistically significant and had a *β* = −1.151 effect on discharge FIM (*P* = 0.014); and specialist referrals was not statistically significant with a *β* = −2.776 effect on discharge FIM (*P* = 0.079).

### 3.3. Living Conditions

**Figure 2:**
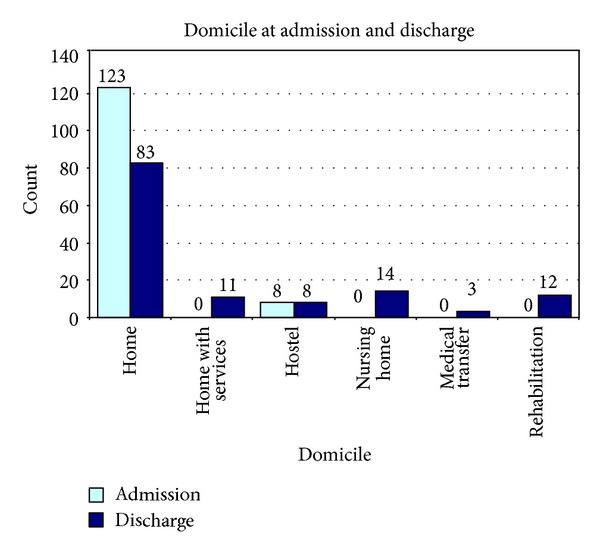
Overall patients' domicile at admission and discharge.

Prior to admission, 123 (94%) of participants were residing in their own homes; 8 (6%) of patients were residing in low-care residential facilities, that is, a hostel, and no patients came from high-care residential facilities, that is, nursing homes (Figure 2).

At discharge, 68% (83/123) of patients returned to their original residence with no changes to personal independence; 9% (11/123) of patients returned home with new (additional) services such as home and community care package (HACC), community aged care packages (CACP), or extended aged care at home packages (EACH); 11% (14/123) of patients entered high-care nursing homes; 9% (12/123) of patients entered rehabilitation; and 2% (3/123) of patients were transferred back to their medical team due to deterioration (Figure 2). There were no deaths during this period.

Changes in domicile at discharge reflect an increase in the dependency on either the health or social-welfare system. The relationship between frailty status at admission and the change in domicile from admission to discharge was examined. A chi-square test for independence was used on change in dependency and frailty status. The chi-square value was *X* ^2^ = 22.442 with df = 2, was significant at the *P* < 0.001 level of significance. Hence, we conclude that it is unlikely that these variables are independent of one another, thus inferring that frailty status has an influence on a patient change in dependency upon discharge.

Table 3 supports this inference by illustrating the movement of patients based on frailty status (pre-frail, frail, or severely frail) of individuals admitted to the GEM for treatment. 62% (23/37) of severely frail patients compared with 71% (44/62) of frail and 50% (16/32) pre-frail individuals were discharged home representing a maintenance of personal independence status; therefore, 38% (14/37) of severely frail compared with 29% (18/62) of frail individuals and 50% (16/32) of pre-frail individuals experienced reduced personal independence after intervention at the GEM.

## 4. Discussion

Previous studies have elucidated age-related patterns of disease presentation, treatment approaches, survival, quality of life, impact of comorbidities, and functional outcomes. A number of recent studies [18–22] and ongoing data bases are being utilised to focus on frailty and functional association. Geriatric assessment approaches have been studied in a number of randomised and controlled studies [12] and work is now concentrating on the application of comprehensive geriatric assessment tools in the evaluation and treatment of older patients. The geriatric evaluation and management (GEM) model involves CGA as well as multidisciplinary management and rehabilitation. Our study aimed to assess the efficacy of a GEM intervention in a frail population and furthermore demonstrated the ability to use the established functional independence measure tool on admission to enable practitioners to apply targeted treatment pathways to effectively improve functional gain based on frailty severity. Geriatric rehabilitation is becoming an increasingly important part of health care provision for the frail older population in the coming future [23, 24]. Multidisciplinary teams in GEM model provide complex intervention to modify the accumulated deficits in the frailty syndrome and improve functional outcomes, and they also appear to increase patient satisfaction with care [7, 11, 22, 25].

Our study found that patient frailty status was inversely correlated with functional independence when using the FIM tool. Frailty also showed stronger correlation with resource utilisation and residential admission. A study investigating the outcomes of older postoperative patients demonstrated that patients with high EFS scores were predisposed to further complications and had a lower chance of being discharged home after surgery [26]. Our study is among the few [18, 19, 27] that demonstrates an association of frailty with functional outcome in a rehabilitation setting. Review of the literature reveals that the frailty indexes are used to predict adverse outcome in the older persons [28] and FIM scale measures a patient's progress and rehabilitation outcomes [19]. We acknowledge that these two tools have some similar domains which can confound the results. However, given that the correlation between frailty and functional outcome might render the confounding error to be negligible. It should be recognised that most of the frailty indicators currently in use have similar issues when using with FIM [29].

The majority of our sample was described as “frail” as per assessment with the EFS. This somewhat resembles other study cohorts previously reported in rehabilitation settings [19]. The frailer group in our study gained better function compared to the other groups. Screening and assessment of frailty are increasingly recommended components of the assessment of older people [19, 30]. We measured frailty using the EFS prior to the patient being discharged. Ideally the baseline premorbid frailty should be performed either in the community or prior to the hospital admission. A patient's frailty score is known to be changeable over time with different interventions [22]. However, our study's population was enrolled from different inpatient units; therefore, we were limited in our ability to get the real baseline FS. It was hypothesised that FS measured prior to discharge would be indicative of the patient's baseline FS. It is generally accepted that assessment of older people is a complex process; vast diversity of instrumental ADLs, baseline function; adherence to the therapy, duration of stay in the rehabilitation unit, and reason of current hospitalisation can all contribute to the functional change of an older person. Frailty assessment is not, of course, an end in itself. Its fundamental purpose is to establish and carry out treatment goals.

Multidisciplinary interventions improved functional abilities and mental well-being of vulnerable older people in a rehabilitation setting [31]. The results of our GEM intervention showed a mean functional improvement across all frailty status groups. The meta-analysis by Stuck et al. [32] for inpatient geriatric care revealed comprehensive geriatric assessment (CGA) and management is effective in improving survival and function in older people [23, 32]. Although careful patient targeting has been advocated as a method of improving the outcomes of services for older persons in GEM care, wide variations in admission criteria and status have led to inconsistent results in functional gain [23].

While in rehabilitation frail older persons often have active medical problems and comorbidities that require close medical management. In three of the Stuck et al. cohorts, some patients had their rehabilitation course complicated by medical illnesses and were transferred back to acute medical care. In our study, 41% (54/131) of patients experienced a one-off medical complication, 9% (12/131) of patients required two external interventions, and 2% (2/131) of patients required 3 or more external specialist consultations to deal with additional medical complications. Furthermore, two of our patients with oncological problems had unexpected functional decline during the course of rehabilitation which resulted in a large decline in the discharge FIM and impaired the mean functional gain of the group. The spectrum of frailty symptoms in older patients is often multifaceted and requires a multidisciplinary approach to the treatment, management, and rehabilitation process [23]. Studies of geriatric rehabilitation have reported similar issues with their patients. Our results showed that regardless of frailty status on average the patients admitted to the GEM experienced functional improvement. GEM intervention is a short stay unit to assess consistency across all frailty groups. Hence, we did not assess whether severely-frail older patients would benefit from longer periods of rehabilitation. The tailoring of the GEM intervention for pre-frail and severely frail individuals might achieve greater clinical effectiveness and needs further investigation.

Our results demonstrated functional improvement after GEM intervention across the frailty groups. Although the standard deviation of the FIM at discharge across the frailty groups overlaps, the range of the standard deviations is become tighter about the mean in functional gain at discharge across frailty status relative to the admission levels. From this we can infer that the GEM intervention is worthy of further investigation to determine the effect of the program on functional improvement in older patients based on frailty status. Frailty dramatically increases healthcare costs, greatly adding to the burden of individuals, families, and society. The healthcare expenses of frail older people are five times those of non-frail elders [5]. In this study, cohorts with higher frailty scores had utilised more institutional services on discharge or needed an increased length of stay by transferring their care to other rehabilitation settings. Interestingly, the smallest proportion of patients discharged to nursing homes were the severely frail group. Another finding from this study was the higher proportion of severely frail patients who were discharged to hostel or home with services representing a reduction in personal independence and an increase in resource utilisation to the healthcare system in the short term. We speculate that the mechanism underpinning these findings could be either the patients preference to return to their homes or that the patients had a partially reversible medical condition, such as post-fracture or joint replacement surgery.

Prior studies have found that frailty and subsequent hospitalisation are a marker of acute illness and strongly associated with onset of reduced independence in activities of daily living [33]. In a multicentre prospective cohort study of 448 hospitalised patients [34], it was identified that the three patient characteristics that were independent predictors of functional decline in the development cohort were increasing age, lower admission Mini-Mental Status Exam scores, and lower preadmission independent activities of daily living function.

In our study population, women were in majority, but no significant gender differences were found in either frailty score or functional improvement. Apart from the fact that age gave different results, this could confirm that older people (age > 80) are more frail compared to patients less than 80 years of old, as previously reported [21, 35]. The work of Fried et al. [21] supports these findings by reporting that those who were frail were older. However, they found females were frailer than males. The increase in life expectancy has resulted in a large segment of the population being over 80 years of age who are vulnerable to medical events and at increased risk of hospitalisation [30, 36]. Two-thirds of our study population was above 80 years old.

A limitation of this study was the inclusion and exclusion criteria. By excluding those patients who suffered a stroke and/or those with severe cognitive impaired group, we diminished the generalisability of our findings. We also acknowledge that there is an overlap between EFS and FIM, and as such our results may have been confounded. However, further investigation is required to determine the severity of confounding based on the similarity of the tools. Another limitation of this study was that frailty was measured prior to discharge and not at their “frailest” on admission. This would have overestimated the patients' baseline frailty score and understated the benefit of the GEM intervention and the functional improvement within the sample. The absence of a control group makes it difficult to assess the effectiveness of the intervention by the GEM unit and requires further investigation. Additionally, a larger sample size would have reduced the chance effects between groups, given a more robust analysis, and increased the generalisability of the analysis as the small numbers per group could have introduced type 2 statistical errors.

Given that this was a case cohort study to assess the effectiveness of the GEM intervention on a patient's functional gain, we focussed on frailty, functional improvement, and discharge destination as outcomes. We limited the impact of the study by not collecting data on the cost of a patient's resource utilisation for an in-hospital stay and the economic burden of disease associated with a loss of personal independence and a change in their discharge destination.

## 5. Conclusion

Based on the results of this study we can conclude that frailty assessment has a prognostic value with regard to functional outcome, institutionalisation, and resource utilisation. Wider findings corroborate on the use of frailty score components as a tool to determine a patients suitability for rehabilitation. Additionally, the comprehensive short stay geriatric rehabilitation unit was shown to provide functional improvement in patients originally admitted and treated by medical or surgical specialities. However, the challenge in research translation of these findings, is to determine how best to use frailty assessment tools to augment the current patient assessment process.

Our study is limited because of the poor sample size and loss of generalisability and because its results must be interpreted within the context of its design. This research illustrates that for those older patients who are referred to a rehabilitation setting after experiencing an acute decline in function during an inpatient stay and/or after surgery in a tertiary hospital, frailty scores can be used to aid in setting appropriate rehabilitation and discharge goals. Patients should not be excluded from GEM care on the basis of their frailty score, as even the severely frail group did benefit functionally.

## Figures and Tables

**Table 1:** Demographics, frailty categories, and the presence of geriatric syndrome.

Demographics		Frailty status					
	Overall (*n* = 131)	Pre-frail		Frail		Severely frail	
		Count (%)	Mean (SD)	Count (%)	Mean (SD)	Count (%)	Mean (SD)
Gender							
Male	51	16 (31%)		21 (41%)		14 (27%)	
Female	79	16 (20%)		40 (51%)		23 (29%)	
Age group							
Age <80	44	20 (45%)	72.6 (4.6)	17 (39%)	74.8 (4.9)	7 (16%)	73.6 (5.9)
Age >80	83	12 (14%)	84.0 (2.0)	44 (53%)	87.7 (4.9)	27 (33%)	89.7 (5.9)
Domicile at admission							
Home	123	32 (26%)		57 (46%)		34 (28%)	
Hostel	8	0 (0%)		5 (63%)		3 (38%)	
Frailty risk factors							
Geriatric syndrome	116	28 (24%)		52 (45%)		36 (31%)	
Falls	97	19 (20%)		45 (46%)		33 (34%)	
Dementia	61	3 (5%)		27 (44%)		31 (51%)	
Other symptoms	63	6 (10%)		27 (43%)		30 (48%)	
Specialist referrals							
0	64	15 (23%)		30 (47%)		19 (30%)	
1	53	14 (26%)		27 (51%)		12 (23%)	
2	12	3 (25%)		3 (25%)		6 (50%)	
3	2	0 (0%)		2 (100%)		0 (0%)	

*Rehabilitation—patients requiring a lengthy period of treatment were transferred to a rehabilitation facility.

**Table 2:** Comparing admission, discharge, and difference in functional improvement across frailty status.

Frailty status	Admission FIM	Discharge FIM	Difference in FIM
	Mean (±SD)	Mean (±SD)	Mean (±SD)
Pre-frail	95.7 (±13.7)	105.7 (12.3)	9.9 (6.9)
Frail	84.3 (±19.3)	96.7 (17.1)	12.4 (11.1)
Severely frail	70.2 (±20.1)	80.73 (22.8)	10.5 (20.9)

**Table 3:** Frailty status by discharge domicile cross-tabulation.

	Discharge domicile						
	Home	Home with services	Hostel	Nursing home	Medical transfer	Rehabilitation	Total
	*n* (%)	*n* (%)	*n* (%)	*n* (%)	*n* (%)	*n* (%)	*n* (%)
Pre-frail (*n* = 32)	16 (50%)	1 (3%)	1 (3%)	5 (16%)	2 (6%)	7 (22%)	32 (24%)
Frail (*n* = 62)	44 (71%)	6 (10%)	3 (5%)	6 (10%)	0 (0%)	3 (5%)	62 (47%)
Severely frail (*n* = 37)	23 (62%)	4 (11%)	4 (11%)	3 (8%)	1 (3%)	2 (5%)	37 (28%)

Total	83 (63%)	11 (8%)	8 (6%)	14 (11%)	3 (2%)	12 (9%)	131 (100%)
